# LivWell: a sub-national Dataset on the Living Conditions of Women and their Well-being for 52 Countries

**DOI:** 10.1038/s41597-022-01824-2

**Published:** 2022-11-22

**Authors:** Camille Belmin, Roman Hoffmann, Mahmoud Elkasabi, Peter-Paul Pichler

**Affiliations:** 1 Social Metabolism and Impacts, Potsdam Institute for Climate Impact Research, Member of the Leibniz Association, Potsdam, PO Box 60 12 03, 14412 Germany; 2 Department of Cultural History & Theory and Department of Social Sciences, Humboldt University Berlin, Berlin, Unter den Linden 6, D-10117 Germany; 3 International Institute for Applied Systems Analysis, Wittgenstein Centre (IIASA, OeAW, University of Vienna), Vienna, Austria; 4 Vienna Institute of Demography (OeAW), Wittgenstein Centre (IIASA, OeAW, University of Vienna), Vienna, Austria; 5 RTI International, Washington, USA

**Keywords:** Developing world, Sustainability, Environmental health, Energy access

## Abstract

Data on women’s living conditions and socio-economic development are important for understanding and addressing the pronounced challenges and inequalities faced by women worldwide. While such information is increasingly available at the national level, comparable data at the sub-national level are missing. We here present the LivWell global longitudinal dataset, which includes a set of key indicators on women’s socio-economic status, health and well-being, access to basic services and demographic outcomes. It covers 447 regions in 52 countries and includes a total of 265 different indicators. The majority of these are based on 199 Demographic and Health Surveys (DHS) for the period 1990–2019 and are complemented by extensive information on socio-economic and climatic conditions in the respective regions. The resulting dataset offers various opportunities for policy-relevant research on gender inequality, inclusive development and demographic trends at the sub-national level.

## Abstract (summary)

**Table:** 

Measurement(s)	human living conditions
Technology Type(s)	data transformation

## Background & Summary

Longitudinal macro-level data on demographic and socio-economic trends are an important source of information for researching and monitoring human development worldwide, for example, related to poverty eradication, advances in education, or improvements in living conditions. While extensive data are available at the national level, e.g. through the World Bank open data repository^1^, only limited comparable data exist at the sub-national level. Such data are particularly important for examining the often significant differences between regions within countries. Information on regional heterogeneity also enables better targeting of policy efforts and supports inclusive policy planning and implementation.

In particular, there is a dearth of comparable sub-national data that accurately reflect women’s living conditions and the specific gendered challenges they face^2^. For example, national data on domestic violence (i.e., the proportion of women subjected to physical and/or sexual violence by a current or former intimate partner) is available for 106 countries in the World Bank open data repository, but the information for each country is available only for one year, making it difficult to explore trends and underlying drivers of change.

Development does not benefit all parts of a population equally and women and girls often face disadvantages and discrimination. High-quality longitudinal data on women’s status and living conditions, both at the national and sub-national levels, are therefore critical to understanding how improvements can be achieved in an inclusive and sustainable manner. The Global Data Lab provides selected sub-national data on women^3^, including in form of the subnational Gender Development Index (SGDI). Here, we build on these efforts, further extending the available range of sub-national indicators with a particular focus on issues related to gender equality, women’s well-being and key demographic outcomes.

**Fig. 1:**
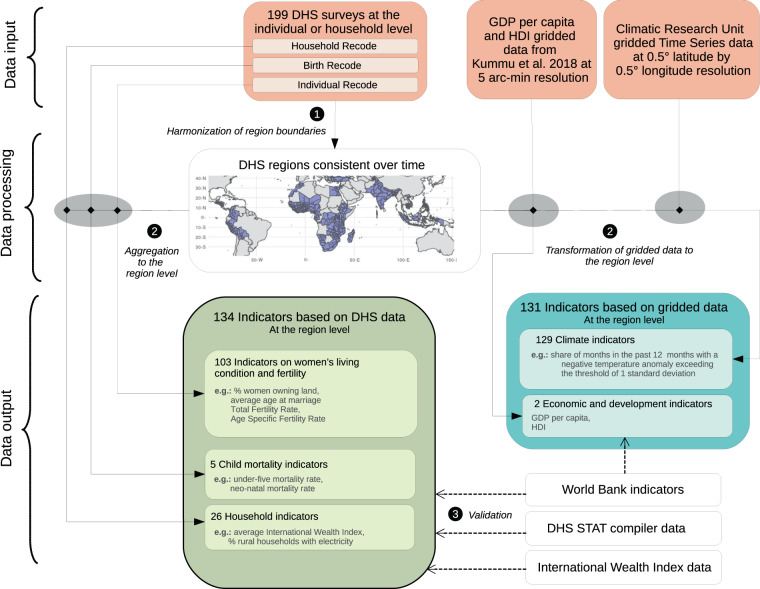
Flowchart representing the data processing steps to obtain LivWell. Orange: input data; green: indicators based on DHS data; blue: indicators based on gridded data; white: validation data.

In this article, we present LivWell^4^: a global longitudinal dataset at the sub-national level, which is mainly derived from Demographic and Health Survey (DHS) data^5^ (https://dhsprogram.com/data/). LivWell is based on the answers of millions of women and collected in 199 DHS surveys in 52 countries. The microdata were aggregated to the sub-national regional level (geo admin 1). The resulting macro-level dataset covers 447 harmonized sub-national regions over a 30-year period from 1990 to 2019. It includes 114 indicators on women’s status and wealth, education, household characteristics, (reproductive) health, fertility and infant health (Fig. 1, Supplementary Table 1). In addition, we included 20 indicators on domestic violence and decision-making power which are particularly difficult to obtain from other sources.

Our dataset provides added value through the extensive harmonization of region boundaries. Over the past decades, changes in the DHS administrative sub-divisions frequently occured, which limits the comparability over time. For the construction of the sub-national data, we have manually harmonized the DHS regions by accounting for changes in countries’ administrative sub-divisions based on the IPUMS International^6^ geographic files (https://www.idhsdata.org/idhs/) and the DHS Spatial Repository^7^ (http://spatialdata.dhsprogram.com/home/). IPUMS International has made considerable efforts to harmonize DHS regions. Yet, only 28 out of 90 DHS countries were harmonized so far, limiting the possibility to analyze data at a large geographical scale over time. In our manual harmonization, we matched the regional boundaries of an additional 24 countries, to derive longitudinal information about regional changes and trends.

Using the spatial boundaries of the harmonized regions, we complemented the aggregated DHS data with additional external information about the climatic and socio-economic conditions in the regions. As climatic factors, we obtained monthly information on temperature and precipitation from the Climatic Research Unit of the University of East Anglia^8^ which we used to identify anomalies and extreme events in the years prior to the DHS data collection. As external socio-economic indicators, we included information on the regional Gross Domestic Product (GDP) per capita and Human Development Index (HDI), which are based on Kummu, Taka, and Guillaume (2018)^9^. In addition to the dataset, we have created the companion *R* package *livwelldata* that makes it easy to use the dataset in R and subset it by selecting indicators of interest. The package and instruction can be found on the git repository of the *R* package: https://gitlab.pik-potsdam.de/belmin/livwelldata.

The resulting dataset offers a variety of opportunities for policy-relevant analysis by enabling researchers to explore demographic trends and underlying drivers of socio-economic developments and changes at the sub national level with a focus on women’s living conditions and well-being. The data could be used, for example, to study the role of policy interventions for socioeconomic and demographic outcomes, the relationship between access to household assets (e.g. electricity, electric appliances) and women’s decision power, the effect of climate change on (reproductive) health^10^, fertility, or household wealth, or the role of environmental and socioeconomic conditions for gender-based violence^11^. A preliminary version of the dataset was recently used to study the effect of access to modern energy on fertility and reproductive choices in 44 countries^12^.

## Methods

### Overview

**Fig. 2:**
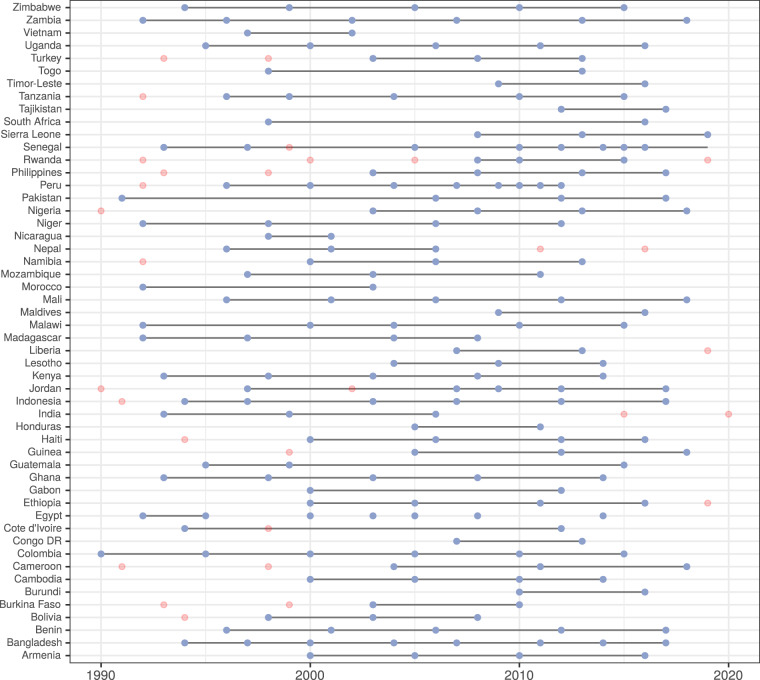
Countries and waves of DHS surveys represented in LivWell. The blue dots correspond to waves represented in LivWell, the pink dots correspond to waves that had to be excluded from LivWell because it was not possible to harmonize the regions.

The LivWell dataset^4^ consists of 265 indicators, which capture women’s living conditions as well as the broader socioeconomic, demographic and environmental conditions in a region. It covers 447 harmonized sub-national regions in 52 countries from 1990 to 2019 (Fig. 2 and Supplementary Table 7). To construct the dataset, we used information from 199 DHS surveys as data sources, which we combined with climate data from the Climatic Research Unit (CRU) of the University of East Anglia^8^, and socio-economic background data from Kummu *et al*.^9^ (Table 1).

**Table 1:** Data sources used in the pre-processing steps and validation.

Data	Used for	Temporal resolution	Spatial resolution	Years	Source
199 DHS survey	LivWell dataset	Every 5, 3 or 1 year	—	1990–2019	ICF^5^
Climatic Research Unit gridded Time Series data	LivWell dataset	Monthly	0.5 deg × 0.5 deg	1990–2019	Harris *et al*.^8^
Gridded global dataset of HDI and GDP	LivWell dataset	Yearly	10 km × 10 km	1990–2015	Kummu *et al*.^9^
DHS STAT compiler data	Validation	Every 5, 3 or 1 year	Region level	1990–2019	ICF^13^
World Bank data	Validation	Yearly	Country level	1990–2019	World Bank^1^
The subnational human development database	Validation	Yearly	Country level	1990–2019	Smits and Permanyer^3^

To construct the dataset, the DHS microdata were aggregated to the sub-national level and combined with gridded data on the climatic and socioeconomic conditions in the regions. The derived sub-national regional data were validated using data derived from the DHS STAT compiler^13^ (https://www.statcompiler.com/en/), the World Bank open data repository^1^ (https://data.worldbank.org/) and the subnational human development database^3^ (Table 1). The LivWell dataset^4^ is an expansion of a dataset that was originally developed for an earlier study on access to modern energy and fertility^12^. The earlier version of the dataset covers 42 countries, 403 sub-national regions and 27 indicators.

The LivWell dataset consists of 5 groups of indicators (Fig. 1 and Supplementary Table 1 and 2 for a full list of indicators). The first group of indicators is based on individual level DHS data (103 indicators) and contains information on women’s living conditions, decision making power, reproductive health, fertility, and issues related to domestic violence. The second and third group of indicators are also based on DHS data and reflect composite measures of child mortality and household wealth. The fourth group of indicators includes regional socio-economic indicators (HDI and GDP per capita) derived from external gridded data provided by Kummu *et al*.^9^. Finally, the fifth group of indicators reflects the environmental and climatic conditions in a region and is derived from the gridded climate data provided by the CRU of the University of East Anglia.

In addition to the raw version, we provide a version of LivWell with linearly interpolated data. As DHS surveys are collected in every country on average only every five years, the original version of LivWell entails gaps between the years of data collection. With the interpolation we create a seamless yearly dataset that allows for the analysis of trends at a finer temporal resolution.

All steps of the data processing and aggregation were carried out in *R*^14^, with the exception of harmonization of the regional boundaries over time, and the harmonization of four DHS variables, which both required a manual step. For each group of indicators, a corresponding *R* function transformed the raw data into the final indicators at the sub-national level. To interpolate the data, we used the *R* package *imputeTS*^15^. Figure 1 summarizes the different data processing steps. The harmonization of the regional boundaries was central for the aggregation and the computation of the indicators, and is described in detail together with the other processing steps below.

### Data sources

The LivWell dataset was compiled from three main data sources (Table 1) which were combined at the sub-national regional level, providing comprehensive information on the demographic, socioeconomic and environmental conditions and trends in a region.

The Demographic and Health Survey (DHS)^5^ represents the primary source of data. Since its creation in 1986, the DHS Program has collected hundreds of surveys in over 90 low- and middle-income countries, focusing on population issues and health. The DHS Program typically adopts a two-stage cluster sampling design that ensures representativeness of the data at the national and sub-national level^16^. Most DHS variables are the same across countries and time, enabling the comparison over space and time. LivWell includes 52 countries out of the 90 countries covered by DHS. Countries had to be excluded either because they had only one DHS wave or because the harmonization of their regions over time was not possible. In addition, two surveys, India 2015 and Turkey 1998, had to be excluded because it was not feasible to load the files on our system. It is possible, however, to calculate the indicators in LivWell also for single DHS survey using the tutorial *adding_country_livwell.Rmd* on the git repository of this article.

For each DHS survey, information is collected using different types of questionnaires. A household questionnaire is used for general questions about the characteristics of the household and its usual residents and visitors. Adult members of the households are then interviewed using a women’s or men’s questionnaire collecting information on a range of topics including fertility, mortality, family planning, marriage, reproductive health, child health and nutrition. DHS data are also a key source of information on domestic violence in low- and middle-income countries. Many recent DHS surveys include a domestic violence module that collects information on women’s experiences with physical, sexual, or emotional violence in their lifetime and during the past 12 months.

To construct the LivWell dataset, we relied on data collected using the DHS women’s and household questionnaires. The information provided in these two questionnaires is typically stored in several DHS datasets that are accessible for data users. We used the women dataset (or Individual Recode), the household dataset (or Household Recode) and the birth dataset (or Birth Recode). The microdata collected among women allow us to gain a unique picture of the status of women and their livelihoods around the world. The data collected at the household level provide additional insights on wealth levels and disparities, housing characteristics, and household demographic composition, which shed further light on the demographic and socioeconomic conditions and trends in an area.

Like other survey-based data sources, the DHS data are prone to sampling and non-sampling errors. The latter type of errors is inevitable in population surveys. These errors can be due to non-response, coverage and measurement issues. The DHS implements different procedures before, during and after the data collection to reduce the effect of these errors^17^. To account for sampling errors, the LivWell dataset provides standard errors for all indicators, which allows users to gauge the uncertainty of individual data points.

As a second data source, we used the global gridded data compiled by Kummu *et al*.^9^ to obtain measures for the socioeconomic development level of the sub-national regions. This dataset provides information on GDP per capita in purchasing power parities and HDI, for the whole world at 5 arc-min resolution for the 25-year period of 1990–2015. To obtain the grid level datasets, the authors made use of available sub-national data whenever possible, combined with national data. Temporal interpolation and extrapolation approaches were used to fill missing values over time (see subsection *Climatic Indicators*). We combined this gridded data with the spatial shapefiles of the regional boundaries to calculate average GDP per capita and HDI level for the DHS regions included in our data.

To obtain information about the climatic conditions and extreme events in the DHS regions, we used data from the CRU of the University of East Anglia (CRU TS v4.05)^8^ as a third data source. The CRU data are provided on a 0.5° latitude by 0.5° longitude grid over all land domains of the world except Antarctica. The data are based on interpolations from weather station observations and provide information on monthly temperatures and precipitation. In addition, we used data from the Global SPEI database^18^, which provides information on the Standardized Evapotranspiration Index (SPEI) at a monthly level. The database is based on monthly precipitation and potential evapotranspiration data from the CRU, again using a 0.5° spatial resolution.

The LivWell dataset can be easily expanded by adding information from other spatial datasets following the procedures outlined later in this section (subsections *Regional socioeconomic indicators* and *Climatic Indicators*) and the code located on the data record^4^ or on the git repository (file *create_dataset.Rmd*, section 7). With the increasing availability of high resolution spatial datasets, users can answer a broad range of research questions by combining LivWell with other data sources. For example, gridded data are now available at a high resolution for global population counts^19^ and density^20^, built up land^21^, and nighttime lights, which have been proposed as a proxy for electrification and economic development. In addition to the CRU-based climate indicators provided in the LivWell dataset, a number of sources provide access to further environmental indicators, including on global forest loss^22^, terrestrial gross carbon dioxide uptake^23^, water bodies^24^, agricultural production^25^, and disaster locations^26^.

### Harmonization of sub-national regions

**Fig. 3:**
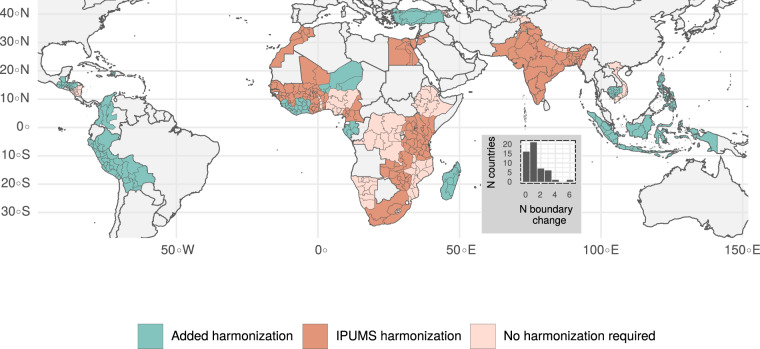
DHS regions in LivWell consistent across time in 52 countries. Inset: number of countries in LivWell by the frequency of regions’ boundary change.

The harmonization of the regions over time was a crucial step to maximize the available number of DHS survey waves per country. The official administrative subdivision of countries changes from time to time, which is also reflected in the Geo Admin 1 regional boundaries of the DHS survey waves. For example, in the surveys used for LivWell, 30% of countries did not change their administrative boundaries, 40% changed their boundaries once, and 13% changed them twice (inset of Fig. 3 and Supplementary Table 7). The goal of the harmonization was to identify the smallest common spatial denominator to allow for comparisons over time, the approach being similar to the IPUMS-DHS International region harmonization.

The harmonization of sub-national regions was done in three steps. First, we collected the region names and indices of all DHS surveys. As a second step, we assigned each region a unique harmonized identifier. For example, one country had 4 regions in the first three DHS waves, but in the last DHS wave the region “R” was split into “R1” and “R2”. The new split-off regions “R1” and “R2” were then assigned the same identifier as the original region “R” and each respondent was assigned the identifier that corresponds to the harmonized region, where she or he resided. When the individual data were aggregated at the sub-national level in the third step, the indicators could then be calculated at the level of the harmonized regions.

To assign the identifiers, we used two sources. First, when available, we used the IPUMS-DHS International region harmonization, which was available for 28 countries. If this information was not available, we used the DHS spatial repository, which shows on an interactive map the region boundaries of DHS data for the different DHS waves. Based on these two sources, we obtained a dataset that maps the raw and harmonized region names and indices of 204 DHS surveys.

**Fig. 4:**
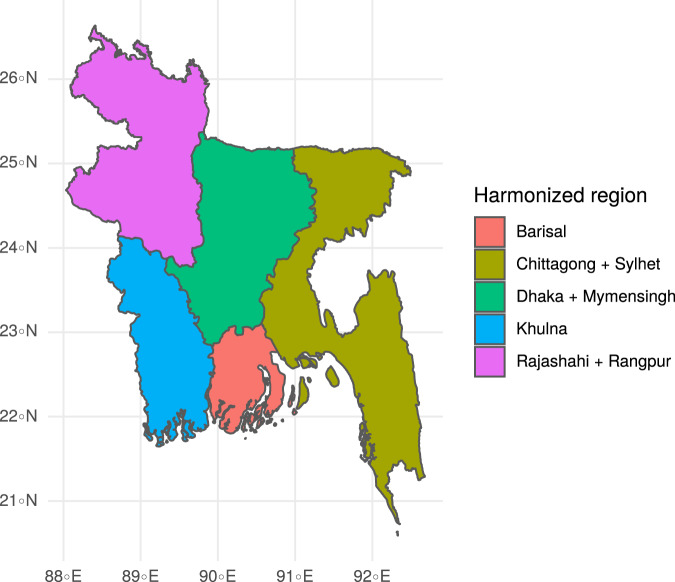
Harmonized DHS regions in Bangladesh. The regions of Dhaka and Mymensingh as well as Rajashahi and Rangpur were combined to a larger spatial entity to account for boundary changes and split-ups of the regions over time.

Once the assignment of harmonized indices was done, we collected the harmonized boundaries using the function *download_boundaries()* from the *R* package *rdhs*^27^ and whenever necessary, merged the region boundaries using the function *st_union()* from the *R* package *sf*^28^ (Figs. 3 and 4). Supplementary Fig. 3 and the file *methodology_harmonization.Rmd* on the data record^4^ and the git repository provide a complete description of the mapping of the raw and harmonized regions and the steps involved in the harmonization.

### Harmonization of DHS variables over time and across countries

Four of the household-level variables used were multi-categorical variables whose categories varied across countries: type of floor, type of toilet, source of drinking water and type of cooking fuel. To create consistent indicators across all countries, we first collected all the possible values of each of these variables across all DHS surveys. Then, we assigned each of these values to a broader category. For variables like type of floor, type of toilet and source of drinking water, we assigned three categories: high, middle and low quality, following the methodology used for the construction of the International Wealth Index (IWI)^29^. For example, for the variable source of drinking water, the value “Piped to dwelling” was assigned to the broader category “high quality drinking water source”, the value “In the Nile” – which was specific to Egypt – was assigned the category “low quality drinking water source” (see also Supplementary Tables 4–6). For the variable reporting the type of cooking fuel used by the household, we assigned two categories: modern cooking fuel and traditional cooking fuel. We included electricity, liquefied petroleum gas, natural gas, kerosene and biogas as modern fuels for cooking. All traditional biomass, namely firewood, charcoal, agricultural crops, animal dung as well as coal, was counted as non-modern cooking fuels.

### Calculation of indicators on women’s living condition

Using the DHS data, we calculated 134 indicators that provide a picture of women’s living conditions and the level of socio-economic development at the sub-national level for 52 countries over 30 years (Supplementary Table 1). To create these indicators for the harmonized regions, the individual DHS data were aggregated to the level of the harmonized sub-national regions (admin 1). Note that for some DHS surveys, the data are also representative at the admin 2 level, but we kept the admin 1 level to remain consistent across surveys.

Three types of indicators were calculated using the DHS data: (i) indicators representing women’s living conditions and fertility, (ii) child mortality indicators and (iii) household wealth indicators. To calculate these indicators, we wrote and the following *R* functions: *calculate_women_indicators()*, *calculate_fertility_indicators()*, *calculate_chmort_indicators()* and *calculate_household_indicators()*, which are located on the data records^4^ as well as the git repository of this article. For the creation of each of these indicators, whenever possible, we followed the methodology outlined in the Guide to DHS Statistics^30^.

In addition, we calculated indicators that are not typically published in the DHS final reports or STAT compiler. For those indicators, we describe their construction in Supplementary Table 1. All indicators were weighted by the relevant survey weight (household or individual weight). We also calculated standard errors for all indicators, using the *R* package *survey*^31^ to account for the stratified two-stage cluster design of the DHS data. For complex indicators like the total fertility rate or child mortality, we used the *R* package *DHS.rates*^32^ to calculate the indicators and their standard errors.

As DHS surveys are typically collected over several months, they can sometimes comprise two calendar years. In these cases, the survey year reported in the LivWell dataset was the year in which most interviews were conducted, as reported by the DHS Program. We also calculated the average month of interview, which was necessary to merge DHS indicators with the climate data (see subsection *Climatic indicators*). Typically, within the same region, interviews were conducted within one to two months time windows (Supplementary Fig. 2).

Prior to aggregating the data at the regional level, the DHS microdata had to be pre-processed. First, within each DHS sample, the region identifiers were harmonized. For this, we merged the survey microdata with the dataset that maps the raw region identifier with the harmonized region identifier (see subsection *Harmonization of sub-national regions*). This way, the microdata could be aggregated to geographical regions that are consistent over time. Second, for the survey files in which the units of analysis are women of fertile age (*Individual Recode* files), we removed cases corresponding to women younger than 15 and older than 49. Third, for DHS variables with country specific variable labels, we added their corresponding harmonized categorization (see subsection *Harmonization of DHS variables over time and across countries*). Last, some of the multi-categorical variables were recoded and simplified into variables with two or three categories. For example, the DHS variable “Main type of cooking fuel” contains several categories: “firewood”, “coal”, “electricity” etc. We created a binary variable taking the value of 1 if the cooking fuel used was modern and 0 otherwise. Based on this information, we derived the composite indicator “percentage of women cooking primarily with modern fuels”.

The first group of DHS indicators obtained reflect women’s living conditions and fertility. These are based on the DHS *IR* files, in which the units of analysis are women aged 15 to 49. While most DHS surveys interview women regardless of marital status, some DHS surveys are restricted to women who had ever been married. These surveys are referred to as “ever-married surveys,” as opposed to the typical “all-women surveys.” To calculate indicators from ever-married surveys that can be compared to indicators calculated from all-women surveys, we followed the recommended DHS approach and applied all-women factors, released in the *IR* datasets, to account for the proportion of women who were never married in a region. See the Guide to DHS Statistics^30^ for more information about using the all-women factors. The fertility indicators were calculated using the *R* package *DHS.rates*^32^

**Fig. 5:**
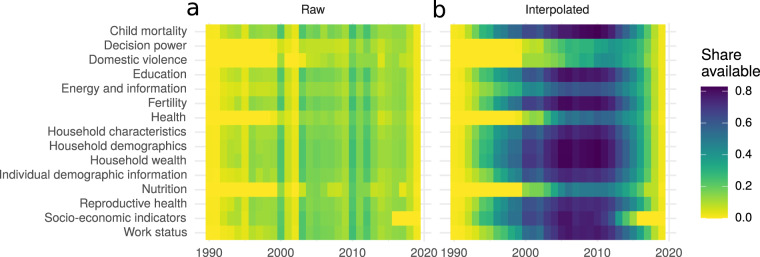
Availability of indicators in LivWell. Share of observation in LivWell available along indicator categories in the raw LivWell version (left) and in the linearly interpolated version (right). Climate data are excluded because they are available for each year.

The fourth and last group of DHS indicators are the household-level indicators about household characteristics, assets and wealth. These indicators are based on the DHS *Household Recode* files. Most of the household indicators are standard and follow the Guide to DHS Statistics^30^, for example the average household size or the proportion of households having a computer. Figure 5 provides the user with an overview of the availability of DHS indicators across different sub-categories of indicators in the raw version of LivWell and in the interpolated version (see also Supplementary Fig. 1 for the availability of countries across different sub-categories of indicators).

### International wealth index

To provide a comparable measure of the level of wealth in a region, the LivWell dataset includes the International Wealth Index (IWI), which was proposed by Smits *et al*.^29^. The IWI is an asset-based index of households’ material well-being, which can be compared across countries and time periods. The construction of the index is similar to other wealth indices, including the DHS wealth index^33^. To construct the measure, we use the results of a principal component analysis (PCA) conducted by Smits *et al*.^29^ considering different variables on households’ asset ownership, access to basic services, and housing characteristics. Based on the PCA, weights were derived for each of these variables (Supplementary Table 3). To conduct the PCA, the authors used data from 165 surveys which were conducted between 1996 and 2011 in 97 low and middle income countries. Of these, 99 were DHS Surveys, 36 Multiple Indicator Clusters Surveys (MHS), 16 World Health Surveys (WHS), and 14 from other surveys and censuses.

To obtain comparable values for the variables “type of floor”, “type of toilet” and “source of drinking water”, we re-categorized the original variables (respectively, v127, v116, v113) into low, medium and high quality categories, using the IWI methodology^29^ (see also subsection *Harmonization of DHS variables over time and across countries* and Supplementary Table 4–6).

To obtain indicators at the region level based on the IWI, we first calculated for each DHS survey and for each household its IWI, using the variable-specific weights. Based on the household information, we then calculated the average, the standard deviation and the Gini coefficient of the IWI for each region.

To calculate the value of the IWI for each household, we followed the procedures described in the original paper by Smits *et al*.^29^. We summed up the different variables on asset ownership and housing characteristics (e.g. quality of toilet facility, number of rooms). Here, each variable was weighted with the first factor of the PCA as described above. The resulting weighted sum of indicators represents the nominator of the wealth index, which was divided by a normalization factor to scale the wealth index to a range from 0 (low wealth) to 1 (high wealth). As some DHS surveys had missing information for some of the wealth variables, we used as normalization factor the maximum possible value the wealth index could take given the variables available. To derive the maximum possible value, we summed up all the positive weights of the non-missing indicators. The formula of the IWI writes:

$$IW{I}_{i}=\frac{25.004470+{\sum }_{item}{d}_{ite{m}_{i}}\ast fPC{A}_{item}}{25.004470+{\sum }_{item}\,fPC{A}_{item}\left(if > 0\right)}$$

with

- *IWI*_*i*_, the value of the International Wealth Index of household *i*;
- the value of 25.004470 reflecting a constant adjustment factor provided in the original paper^29^;
- ${d}_{ite{m}_{j}}$, a dummy variable measuring whether the household possessed an item (1) or not (0). The items are listed in Supplementary Table 3;
- *fPCA*_*item*_, the weight, corresponding to the first factor of the PCA, for a given item. The values are shown in Supplementary Table 3.

### Regional socioeconomic indicators

Based on the gridded global datasets for GDP and HDI over 1990–2015^9^, we obtain the regional socioeconomic background indicators, GDP per capita and HDI, which we combine with the aggregated DHS data. The GDP captures the monetary value of all final goods and services in a region in a year^9,34^. The HDI is a composite index measuring achievements in key dimensions of human development related to health, education, and economic development. The values of the GDP (PPP) per capita are given in constant 2011 international US dollars thus enabling comparisons between years.

To produce the original gridded datasets, Kummu *et al*.^9^ used a combination of national and sub-national data sources. For GDP, they used a previously published sub-national GDP per capita dataset compiled by Gennaioli *et al*.^35^ together with national information from the World Bank Development Indicators database^36^ (https://databank.worldbank.org/source/world-development-indicators/) and the CIA’s World Factbook^37^ (https://www.cia.gov/the-world-factbook/). To collect sub-national HDI data, they relied on various sources, including censuses and UNDP reports for countries outside of Europe as well as the sub-national Eurostat database for countries within Europe. National-level data on HDI was obtained from UNDP^38^ (http://hdr.undp.org/en/data).

For the construction of the datasets, the authors created a seamless administrative raster file covering the global land area. Based on the national and sub-national information available, they constructed yearly datasets using temporal interpolation (thin plate spline approaches) and extrapolation (based on national trends for sub-national, and regional trends for national estimation). For missing data at the national level, information was derived using regional data (see page 3 and the following pages in the original paper^9^ for a detailed description of the procedures).

We used the information on the spatial boundaries of the regions to crop the gridded data and calculate average GDP per capita and HDI values for each of the DHS regions. The boundary information was saved as a multi-polygon geopackage and is available for download together with the other materials on the data record^4^ and on the git repository of the paper. The *R* package *raster*^39^ was used for the data transformation. First, the raster data over time is clipped to the spatial boundary data to obtain a multilayered rasterbrick object containing information on the DHS region each raster grid falls into (either fully or partly). Based on this, an aggregate regional value was derived using the *extract()* function, which returns the mean value of all raster cells whose center lie inside the boundaries of a region (the weight option can be used to also consider partly covered cells). The resulting dataset contains information on a yearly basis from 1990 to 2015. By joining these data to the aggregated DHS data, we obtain information on regional GDP per capita and HDI values for each of the DHS waves until 2015.

The data on the regions’ socioeconomic conditions come with certain limitations, which were reported by Kummu *et al*.^9^ in their article. The accuracy of the data can vary between regions and over time depending on the available source data. Also, many of the data sources used for the construction of the gridded dataset report only average value, which may not reflect the possible heterogeneity within an area. In addition, the availability of source data over time is an issue. Especially the subnational data on HDI were available only for a single year. Changes in subnational HDI in relation to national HDI may hence not be well captured in the dataset. And finally, the interpolation and extrapolation approaches used come with certain assumption and uncertainties, which may affect the results.

### Climatic indicators

Indicators on environmental and climatic conditions in a region were derived from the CRU monthly high-resolution gridded time series dataset (CRU TS 4.05)^8^, and the SPEI Database, which also uses CRU data. The CRU time series is based on global observations from weather stations. The individual observations are transferred to a 0.5° regular grid using angular distance weighting for interpolation (see page 6 in Harris *et al*.^8^ for a detailed description of the interpolation procedures). An overview of climate indicators can be found in Supplementary Table 2.

The SPEI database provides information about drought conditions globally at a 0.5° spatial resolution. It combines information on monthly precipitation and potential evapotranspiration and uses a standardized intensity scale with higher values indicating more humid, and lower values indicating drier conditions. The SPEI can be calculated for different time periods from 1 to 48 months, reflecting the time scale over which the water balance in a region is measured. Here, we provide indicators based on SPEI03 data, as previous research has suggested that a 3-month SPEI is well-suited to monitor drought impacts on vegetation^40^. Users can flexibly adjust the temporal scale by replacing the SPEI03 data with other datasets.

In a first step, we derived gridded information on monthly average temperatures in °C, precipitation in mm, and SPEI03 from the different data sources. In a second step, we once more used the spatial boundaries of the regions to crop the gridded climate data and calculate the mean monthly temperature, precipitation, and SPEI03 per region, following the same procedure as outlined in subsection *Regional socioeconomic indicators*. This gives us a long monthly time-series with climate information from 1900 to 2020.

To derive indicators that can be used in statistical analyses, we calculated a number of composite climate indicators on local climatic conditions, anomalies, and changes over time. We created climate variables for three time windows to reflect different time horizons of climatic influences: The first set of indicators captures climatic conditions 12 months [_12] prior to the measurement of the DHS survey, the second set of indicators considers 36 months [_36] (roughly reflecting the period between two DHS survey waves), and the third set of indicators considers a period of 60 months [_60].

As first composite climate indicators, we calculated the average (_mean), minimum (_min), and maximum (_max) temperature, precipitation, and SPEI03 in a region 12, 36, or 60 months prior to the DHS survey. For precipitation, we additionally calculated the total rainfall in mm for the different time periods considered (_sum).

As a second type of indicators, we calculated standardized anomalies in the region. For this, we calculated the long-run mean and standard deviation for each of the measures over the reference period 1900–2020 in a region *i* for a given month *m*. We then calculated for each month in a year *y* the deviation from the long-run mean and standardized it using the long-run standard deviation (SD) for the entire reference period using the following formula:

$$Anomal{y}_{imy}=\frac{{X}_{imy}-{X}_{im}}{S{D}_{im}}$$

where *X*_*imy*_ represents the temperature, precipitation or SPEI03 in a region *i* in month *m* and year *y*, and *X*_*im*_ and *SD*_*im*_ the average value and standard deviation of *X* for region *i* in month *m* calculated over all years. The derived monthly anomaly measures are standardized and can be interpreted as monthly (positive or negative) deviations from the long-run mean in terms of standard deviations of the local distribution. Based on these monthly values, we calculate the mean temperature, precipitation and SPEI03 anomaly as well as the mean absolute (_abs) anomaly 12, 36, and 60 months prior to the DHS survey. While the first of the two measures averages over both the positive and negative anomalies, the second one takes only the absolute deviation from the mean into account, reflecting the intensity of the fluctuations over time.

In addition, based on the calculated anomalies, we derive a third set of composite indicators, which summarize the share of months [_share] in the past 12, 36, or 60 months, where the climatic conditions have deviated from the long-run mean by 1 SD [_1sd], 1.5 SD [_1.5sd], 2 SD [_2sd] or 3 SD [_3sd], allowing to comprehensively capture anomalies of different intensity. Here, we again distinguish positive anomalies [_anom_p] with a climate value exceeding the long-run mean and negative anomalies [_anom_n] with a value below the long-run mean. This way, we can for example distinguish periods with extreme temperatures above or periods with rainfall below the long-run average. Based on the share measures, it is also possible to calculate the total number of months with an anomaly by simply multiplying the share with the considered time window of 12, 36, or 60 months.

Using the monthly SPEI03 data, we additionally calculate the share of months in the past 12, 36, or 60 months which experienced drought conditions with SPEI03 values below different absolute thresholds. Here, we distinguish between months with an SPEI03 below −1, −1.5, and −2, reflecting different levels of drought intensity. The different types of climatic indicators provide a comprehensive picture of the climatic conditions and anomalies in an area and can be used for a wide range of environment-population analyses.

The climate data were calculated on a monthly basis. In contrast, indicators based on DHS data are reported for one year. When merging the DHS indicators with the climate indicators, we used the average DHS interview month as reference to merge the relevant climate information (see subsection *Calculation of indicators on women’s living condition*). The climate data assigned to the DHS indicators thus accurately reflects the climate conditions that respondents experienced during the 12, 36, or 60 months prior to the interview.

## Data Records

The data and codes to reproduce the dataset are hosted on Zenodo, which is a general-purpose open data repository developed under the European OpenAIRE program and operated by CERN. The data are hosted at the permanent DOI 10.5281/zenodo.5821532^4^. There are three *csv* files and one *gpkg* (GeoPackage) file in the repository. The first file *livwell.csv* contains the LivWell dataset itself with all 265 indicators for 52 countries, 447 sub-national regions over 30 years. The *livwell.csv* file also contains the standard errors for each indicator based on DHS data. The second file, livwell_lin_interpolated.csv contains the Livwell dataset including the linearly interpolated data. The third file *indicators.csv* includes the metadata and description of indicators. The fourth file *harmonized_boundaries.gpkg* contains the harmonized boundaries of the sub-national regions used in our dataset, together with the indices and names of regions. The dataset LivWell may be shared and adapted under the conditions of the CC-BY 4.0 License: https://creativecommons.org/licenses/by/4.0/.

In addition, the records contain a folder *code_livwell* with all the codes and intermediary data to reproduce the dataset or to add new indicators or new surveys. We also included two tutorials: name *adding_indicator_livwell.Rmd*, to add a new indicator, and *adding_country_livwell.Rmd*, to add a new country or survey. However, this is a static version of the code. We encourage users to use the git repository to have the latest version of the code: https://gitlab.pik-potsdam.de/belmin/livwelldata-paper/.

## Technical Validation

**Fig. 6:**
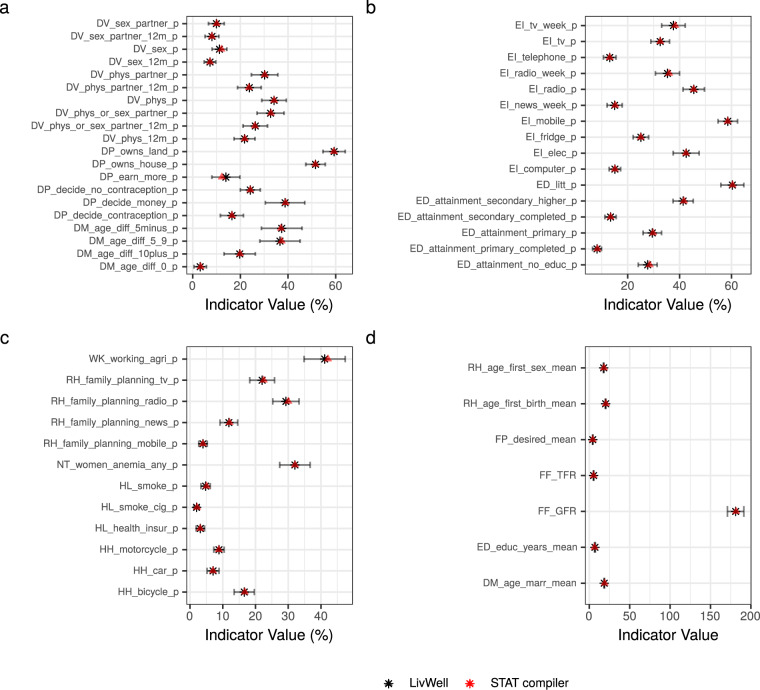
Comparison of 55 indicators in LivWell with the DHS STAT compiler. The comparison is based on 35 DHS surveys. The black star corresponds to the value of the indicators from the LivWell dataset, the red star corresponds to the value of the DHS STAT compiler, and the error bar corresponds to the 95% confidence interval derived from the standard errors of indicators from LivWell.

**Fig. 7:**
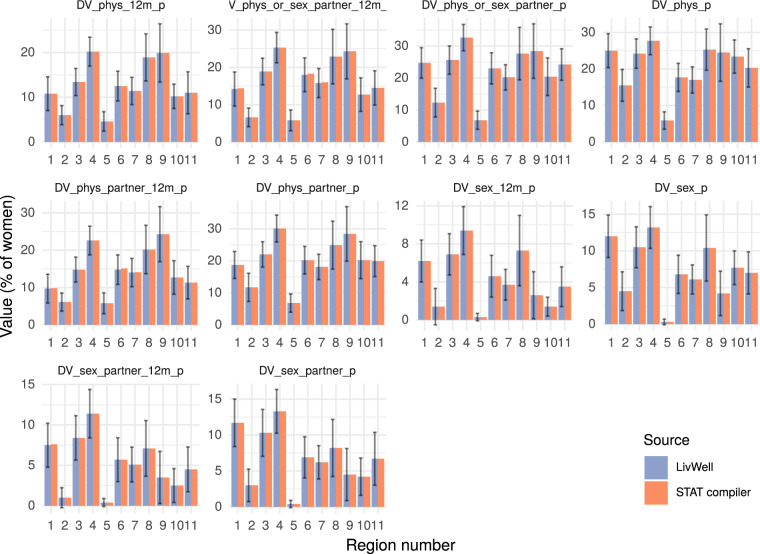
Validation of 10 domestic violence indicators for the DHS survey Ethiopia 2016. In purple are the values of the indicators in the dataset LivWell, in orange are the same indicators extracted from the DHS STAT compiler. For the indicator from LivWell, the error bar represents the 95% confidence interval. Standard errors are not available in the DHS stat compiler.

To validate the LivWell dataset, we proceeded to an internal validation using data from the DHS STAT compiler^13^, and an external validation using data from the World Bank open data repository^1^ and from the subnational human development database^3^ (Table 1). The STAT compiler is a user interface application developed by the DHS Program that allows to visualize indicators based on DHS survey data. Indicators are also available at the sub-national level. If the code to produce LivWell is sound and if the logic to calculate indicators follows the DHS standards, the values of the indicators in LivWell should match the values in the DHS STAT compiler. Since the regions boundaries are not harmonized over time in the STAT compiler, we could only compare the countries for which the definition of regions did not change over time (e.g. Ethiopia or Nigeria). This resulted in 35 comparable surveys, covering 14 countries. We were able to compare 55 out of the 134 indicators based on DHS data, since not all LivWell indicators based on DHS data are available in the DHS STAT compiler. All indicators we compared for the 35 surveys matched the values from the STAT compiler (Fig. 6). We provide a more detailed example of this comparison for 10 domestic violence indicators for the DHS survey Ethiopia 2017 in Fig. 7. For “ever-married surveys” (see subsection *Calculation of indicators on women’s living condition*), the STAT compiler does not produce indicators for all women by applying the all-women factors. Therefore, we were not able to validate the indicators for the ever-married surveys.

**Fig. 8:**
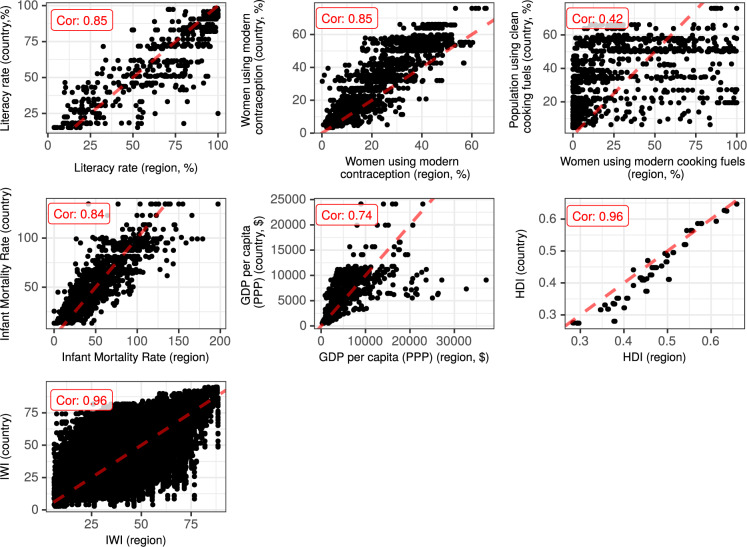
External validation of the LivWell dataset. Comparison between LivWell indicators at the sub-national level (x-axis) and indicators from external data sources at the country level (y-axis). External data sources are the World Bank data and the International Wealth Index from the subnational human development database (Table 1). The red dotted line corresponds to the 1 to 1 ratio.

For some indicators that we were not able to compare with the STAT compiler data, we proceeded to an external validation. To do so, we used country-level comparable indicators from the World Bank open data repository^1^ and from the subnational human development database^3^, and plotted these values against the corresponding values from our dataset at the region level (Fig. 8). We can see the within-country variability for each country, along the x-axis. In addition to the International Wealth Index data, five indicators had comparable definitions with World Bank data, and could be validated through this process.

Overall, the relationship between the country-level indicator and the region-level indicator from LivWell is highly linear, suggesting a good fit. For some indicators, there is a slight difference due to differences in the definition of indicators. For example, for the variable percentage of women using modern contraception, the country level data tend to be higher than the region level data because they are calculated based on married women only (whose contraceptive use tends to be higher than for unmarried women). For the indicator on cooking fuels, the relationship is not as linear as with other indicators because the country level is based on clean cooking fuels (that can include firewood burnt in a clean way), whereas our indicators on modern cooking fuel excludes wood. The GDP indicator from the World bank that had the most available data was in 2017 constant $, while our measure of GDP was calculated in 2011 constant $, which probably explains the lower correlation.

Finally, since not all indicators could be validated through the internal and external validation, we also made sure that all values of indicators included LivWell had realistic summary statistics, e.g. that all proportions range between 0 and 1 and that continuous indicators have realistic averages (Supplementary Table 8–10).

## Usage Notes

The LivWell dataset is provided in *csv* format so it can be easily used in any data processing software. The file with harmonized boundaries is provided as a *gpkg* (Geopackage) file and can be opened and processed using, for example, *R*, *python* or *QGIS*.

We also created a *R* package named *livwelldata* allowing to easily use the dataset in *R*. The package contains four functions. The function *livwell_data()* allows to load the whole dataset or a subset based on a selection of countries, years and/or indicators. It contains a parameter *interpolated* allowing to load the data including interpolation. The function *livwell_indicators()* allows to extract the list of all available indicators. The function *livwell_countries()* allows to extract the list of countries in the dataset. The function *livwell_harmonized_regions()* allows to extract the list of harmonized sub-national regions. The *R* package *livwelldata* as well as the instruction to use it can be found on this git repository: https://gitlab.pik-potsdam.de/belmin/livwelldata.

## Supplementary information

Supplementary Information

## Data Availability

The processing steps to obtain the dataset were carried out in *R* and *Rmarkdown* and are reproducible (except for one step of the harmonization of DHS regions and variables that had to be done manually). All the code is available on the git repository of this article: https://gitlab.pik-potsdam.de/belmin/livwelldata-paper. The source code for the companion *R* package livwelldata is available on the git repository of the package: https://gitlab.pik-potsdam.de/belmin/livwelldata. The following *R* packages were central to the development of LivWell: *tidyverse*^41^, *knitr*^42^, *rdhs*^27^, *DHS.rates*^32^ and *survey*^31^.
