# Supplementary material for: LivWell: a sub-national Dataset on the Living Conditions of Women and their Well-being for 52 Countries
Source: Sci Data. 2022 Nov 22;9:719. doi: 10.1038/s41597-022-01824-2 (PMC9684414; doi:10.1038/s41597-022-01824-2)
Supplement: Supplementary file 1 — Supplementary Information [file 41597_2022_1824_MOESM1_ESM.pdf]

Supplementary Information

LivWell: a sub-national Dataset on the Living Conditions of Women and their Well-being for 52 Countries

Camille Belmin[1,2] Roman Hoffmann[1,3,4] Mahmoud Elkasabi[5]
Peter-Paul Pichler[1]

November 14, 2022

Contents

Supplementary Figure 1 **2**

Supplementary Figure 2 **3**

Supplementary Figure 3 **4**

Supplementary Table 1 **5**

Supplementary Table 2 **12**

Supplementary Table 3 **14**

Supplementary Table 4, 5 and 6 **15**

Supplementary Table 7 **16**

Supplementary Table 8, 9 and 10 **17**

[1] Potsdam Institute for Climate Impact Research, Germany
[2] Humboldt University of Berlin, Germany
[3] International Institute for Applied Systems Analysis, Wittgenstein Centre (IIASA, OeAW, University of Vienna), Austria
[4] Vienna Institute of Demography (OeAW), Wittgenstein Centre (IIASA, OeAW, University of Vienna), Austria
[5] RTI International, USA

Supplementary Figure 1

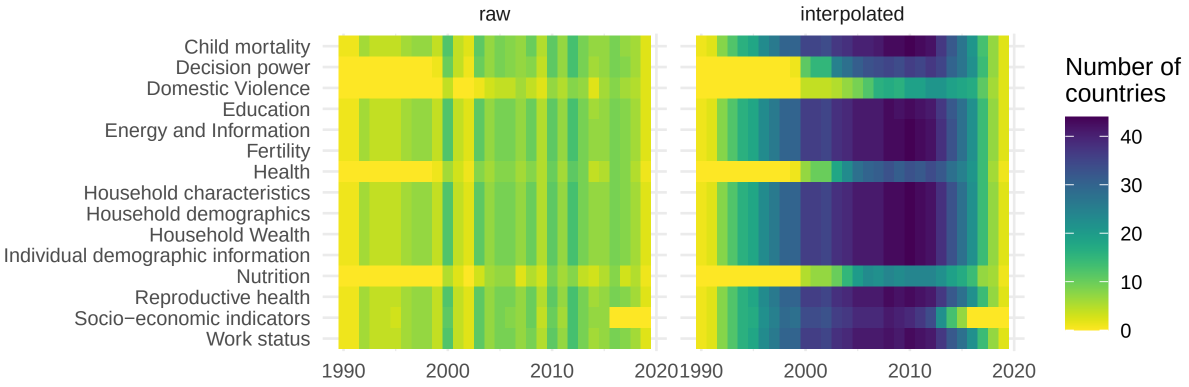

Figure 1: Number of countries available in LivWell along indicator categories in the raw LivWell version (left) and in the linearly interpolated version (right). Climate data are excluded because they are available for each year.

Supplementary Figure 2

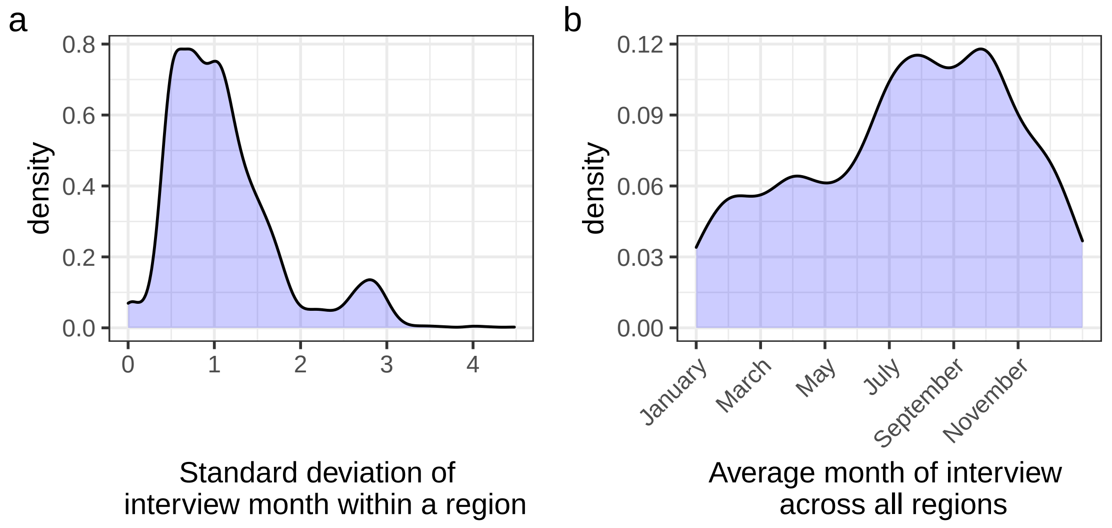

Figure 2: Density plot of the standard deviation of the interview month within a region in LivWell (a) and of the average interview month across all regions in LivWell (b)

Supplementary Figure 3

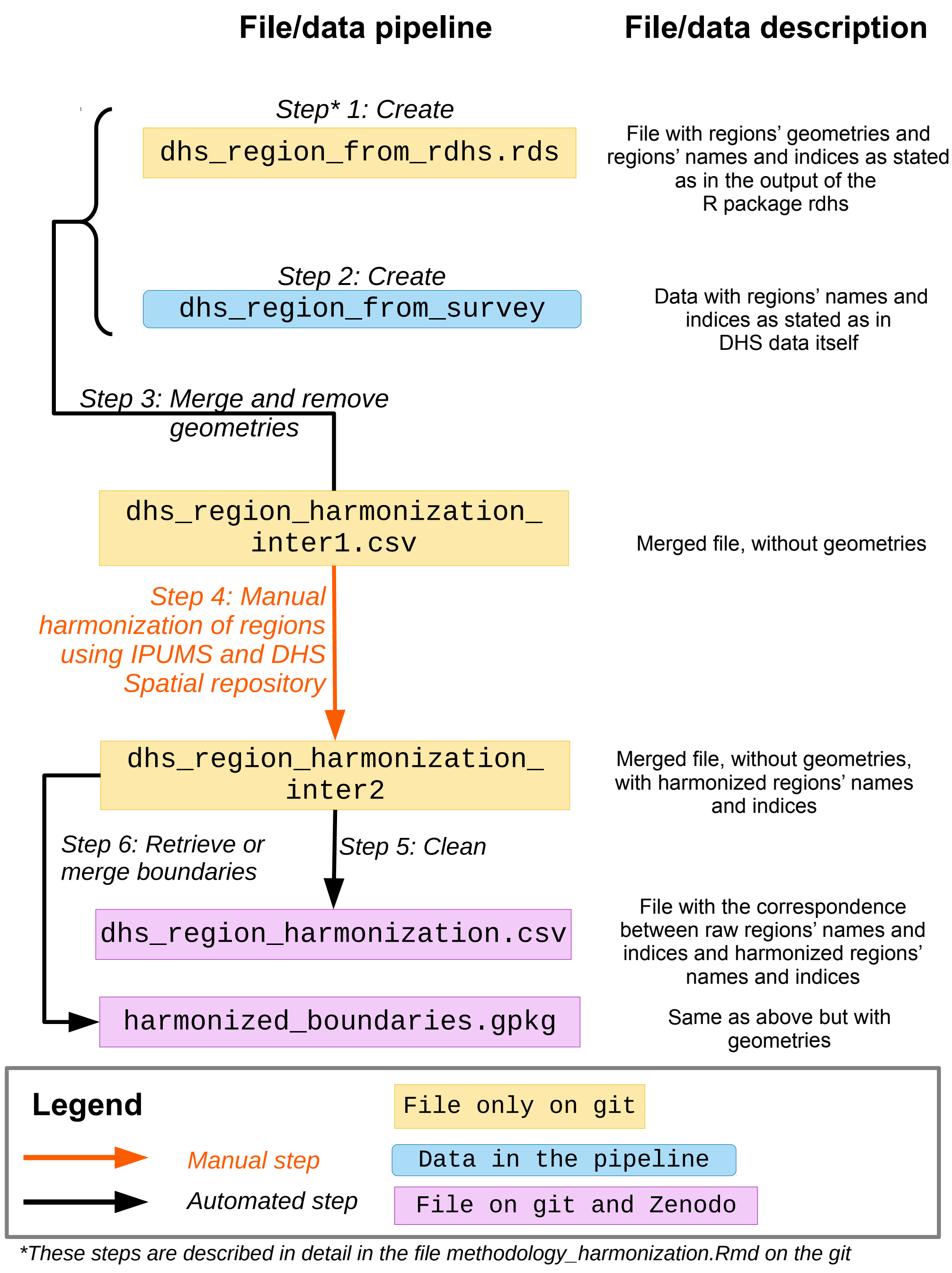

Figure 3: Flowchart representing the data processing steps to obtain the region harmonization file and the corresponding spatial boundaries.

Supplementary Table 1

Table 1: List of indicators based on DHS data present in the database. The indicators for which there is no information on the denominator and nominator were calculated based on the guide for DHS statistics.

Category	Indicator code	Indicator description	Nominator	Denominator or exposure
**Individual demographic information**	DM_age_mean	Average age of women	Not applicable (average)	All women (aged 15-49)
**Individual demographic information**	DM_age_15-19_p	Women in age category 15-19 (%)	Number of women in age category 15-19	Number of women (aged 15-49)
**Individual demographic information**	DM_age_20-24_p	Women in age category 20-24 (%)	Number of women in age category 20-24	Number of women (aged 15-49)
**Individual demographic information**	DM_age_25-29_p	Women in age category 25-29 (%)	Number of women in age category 25-29	Number of women (aged 15-49)
**Individual demographic information**	DM_age_30-34_p	Women in age category 30-34 (%)	Number of women in age category 30-34	Number of women (aged 15-49)
**Individual demographic information**	DM_age_35-39_p	Women in age category 35-39 (%)	Number of women in age category 35-39	Number of women (aged 15-49)
**Individual demographic information**	DM_age_40-44_p	Women in age category 40-44 (%)	Number of women in age category 40-44	Number of women (aged 15-49)
**Individual demographic information**	DM_age_45-49_p	Women in age category 45-49 (%)	Number of women in age category 45-49	Number of women (aged 15-49)
**Individual demographic information**	DM_urban_p	Women living in urban areas (%)	Number of women (aged 15-49) living in urban areas	Number of women (aged 15-49)
**Individual demographic information**	DM_born_rural_p	Women being born at the country side (%)	Number of women (aged 15-49) born at the country side	Number of women (aged 15-49)
**Individual demographic information**	DM_marr_p	Women currently married (%)	-	-
**Individual demographic information**	DM_nvr_marr_p	Women never married (%)	-	-
**Individual demographic information**	DM_age_marr_mean	Female average age at marriage	-	-
**Individual demographic information**	DM_age_diff_mean	Average difference between women and husband/partner, For currently married women aged 15-24	Not applicable (average)	Women currently in an union
**Individual demographic information**	DM_age_diff_10plus_p	Young women (aged 15-24) whose partner is 10 years older than them (%)	-	-
**Individual demographic information**	DM_age_diff_5_9_p	Young women (aged 15-24) whose partner is between 5 and 9 years older than them (%)	-	-
**Individual demographic information**	DM_age_diff_5minus_p	Young women (aged 15-24) whose partner is less than 5 years older than them (%)	-	-
**Individual demographic information**	DM_age_diff_0_p	Young women (aged 15-24) whose partner is the same age as them (%)	-	-
**Household characteristics – Womens level**	HH_women_time_water_mean	Average time to get to drinking water	Not applicable (average)	Women living in households whose source of water is not on premise
**Household characteristics – Womens level**	HH_women_toilet_high_p	Women living in households with high quality toilet facility (%)	Number of women living in households with high quality toilet facility (for the definition of toilet categories see Supplementary Table 5)	Number of women (aged 15-49)

Table 1: List of indicators based on DHS data present in the database. The indicators for which there is no information on the denominator and nominator were calculated based on the guide for DHS statistics. *(continued)*

**Category**	**Indicator code**	**Indicator description**	**Nominator**	**Denominator or exposure**
**Household characteristics – Womens level**	HH__women__toilet__low__p	Women living in households with low quality toilet facility (%)	Number of women living in households with low quality toilet facility (for the definition of toilet categories see Supplementary Table 5)	Number of women (aged 15-49)
**Household characteristics – Womens level**	HH__women__floor__high__p	Women living in a household with high quality floor (%)	Number of women living in households with high quality floor (for the definition of floor categories see Supplementary Table 4)	Number of women (aged 15-49)
**Household characteristics – Womens level**	HH__women__floor__low__p	Women living in a household with low quality floor (%)	Number of women living in households with low quality floor (for the definition of floor categories see Supplementary Table 4)	Number of women (aged 15-49)
**Household characteristics – Womens level**	HH__women__water__high__p	Women living in a household with high quality water access (%)	Number of women living in households with high quality drinking water source (for the definition of toilet categories see Supplementary Table 6)	Number of women (aged 15-49)
**Household characteristics – Womens level**	HH__women__water__low__p	Women living in a household with low quality water access (%)	Number of women living in households with low quality drinking water source (for the definition of drinking water source categories see Supplementary Table 6)	Number of women (aged 15-49)
**Household characteristics – Womens level**	HD__women__size__mean	Average number of household members	Not applicable (average)	Not applicable (average)
**Household characteristics – Womens level**	HD__women__children__mean	Average number of children resident in the household under the age of 5	Not applicable (average)	Not applicable (average)
**Work status**	WK__working__p	Women currently working (%)	Number of women currently working	Number of women (aged 15-49)
**Work status**	WK__working12__p	Women who worked in the past 12 months (%)	-	-
**Work status**	WK__working__paid__p	Women who women who worked in the past 12 months who are paid in their work (%)	-	-
**Work status**	WK__working__agri__p	Women of women who worked in the past 12 months whose occupation is in agriculture (%)	-	-
**Education**	ED__educ__years__mean	Female average/median years of schooling	-	-
**Education**	ED__attainment__no__educ__p	Women with no education (%)	-	-
**Education**	ED__attainment__primary__p	Women with primary education (%)	-	-
**Education**	ED__attainment__primary__compl	Women with completed primary education (%)	-	-
**Education**	ED__attainment__secondary__p	Women with secondary education (%)	Number of women with some secondary education	Number of women (aged 15-49)
**Education**	ED__attainment__secondary__com	Women with completed secondary education (%)	-	-
**Education**	ED__attainment__secondary__higher__p	Women with secondary education or higher education(%)	-	-
**Education**	ED__litt__whole__p	Women who can read a whole sentence (%)	-	-
**Education**	ED__litt__p	Women who are literate (%)	-	-
**Energy and information**	EI__women__elec__p	Women living in an household with electricity (%)	Number of women living in an household with access to electricity	Number of women (aged 15-49)

Table 1: List of indicators based on DHS data present in the database. The indicators for which there is no information on the denominator and nominator were calculated based on the guide for DHS statistics. *(continued)*

Category	Indicator code	Indicator description	Nominator	Denominator or exposure
**Energy and information**	EI__women__tv__p	Women living in an household with tv (%)	Number of women living in an household with a TV	Number of women (aged 15-49)
**Energy and information**	EI__women__radio__p	Women living in an household with radio (%)	Number of women living in an household with a radio	Number of women (aged 15-49)
**Energy and information**	EI__women__fridge__p	Women living in an household with fridge (%)	Number of women living in an household with a fridge	Number of women (aged 15-49)
**Energy and information**	EI__women__telephone__p	Women living in an household with a telephone (%)	Number of women living in an household with a phone	Number of women (aged 15-49)
**Energy and information**	EI__women__modern__cooking__p	Women using modern fuel as main cooking fuel (%)	Number of women cooking with modern fuels (for the definition of modern fuels see main manuscript)	Number of women (aged 15-49)
**Energy and information**	EI__mobile__p	Women owning a mobile phone (%)	-	-
**Energy and information**	EI__internet__week__p	Women using the internet at least one a week (%)	-	-
**Energy and information**	EI__internet__day__p	Women using the internet at least once a day (%)	-	-
**Energy and information**	EI__news__week__p	Women reading a newspaper a least once a week (%)	-	-
**Energy and information**	EI__radio__week__p	Women listening to the radio at least once a week (%)	-	-
**Energy and information**	EI__tv__week__p	Women watching TV at least once a week (%)	-	-
**Decision power**	DP__decide__money__p	Women currently married or in union who were paid cash for their work, who decide alone how their money earned is spent (%)	-	-
**Decision power**	DP__decide__health__p	Women who have the final say on their health care (%)	-	-
**Decision power**	DP__decide__large__purchase__p	Women who have the final say on large household purchases(%)	-	-
**Decision power**	DP__decide__visits__p	Women who have the final say on visiting family or relatives (%)	-	-
**Decision power**	DP__decide__contraception__p	Women deciding alone on their contraception (%)	-	-
**Decision power**	DP__decide__no__contraception__p	Women deciding alone on not using contraception (%)	-	-
**Decision power**	DP__owns__house__p	Women owning a house alone or jointly (%)	-	-
**Decision power**	DP__owns__land__p	Women owning land alone or jointly (%)	-	-
**Decision power**	DP__earn__more__equal__p	Women currently married or in union who were paid cash for their work, earning more or equal than her partner (%)	Women earning more or as much as their partner	Women currently in an union and paid for their work
**Decision power**	DP__earn__more__p	Women currently married or in union who were paid cash for their work, earning more than her partner (%)	-	-
**Domestic Violence**	DV__phys__partner__p	Women who ever experienced physical violence by partner (%)	-	-
**Domestic Violence**	DV__phys__partner__12m__p	Women who experienced physical violence by partner in the last 12 months(%)	-	-
**Domestic Violence**	DV__sex__partner__p	Women who ever experienced sexual violence by partner (%)	-	-

Table 1: List of indicators based on DHS data present in the database. The indicators for which there is no information on the denominator and nominator were calculated based on the guide for DHS statistics. *(continued)*

Category	Indicator code	Indicator description	Nominator	Denominator or exposure
**Domestic Violence**	DV_sex_partner_12m_p	Women who experienced sexual violence by partner in the last 12 months (%)	-	-
**Domestic Violence**	DV_phys_or_sex_partner_p	Women who ever experienced physical or sexual violence by partner (%)	-	-
**Domestic Violence**	DV_phys_or_sex_partner_12m_p	Women who experienced physical or sexual violence by partner in the past 12 months (%)	-	-
**Domestic Violence**	DV_phys_p	Women who ever experienced physical violence since age 15 (%)	-	-
**Domestic Violence**	DV_phys_12m_p	Women who experienced physical violence in the past 12 months(%)	-	-
**Domestic Violence**	DV_sex_p	Women who ever experienced sexual violence (%)	-	-
**Domestic Violence**	DV_sex_12m_p	Women who experienced sexual violence in the past 12 months (%)	-	-
**Health**	HL_smoke_p	Women who smoke (%)	-	-
**Health**	HL_smoke_cig_p	Women who smoke cigarettes (%)	-	-
**Health**	HL_number_cigarettes_mean	Average number of cigarettes in the last 24 hours (% of women who smoke cigarettes	Not applicable (average)	Women who smoke cigarettes
**Health**	HL_smoke5_p	Women who smoke >5 cigarettes in the last 24 hours (%)	Number of women who smoked more than 5 cigarettes in the last 24 hours	Women who smoke cigarettes
**Health**	HL_smoke10_p	Women who smoke >10 cigarettes in the last 24 hours (%)	Number of women who smoked more than 10 cigarettes in the last 24 hours	Women who smoke cigarettes
**Health**	HL_smoke15_p	Women who smoke >15 cigarettes in the last 24 hours (%)	Number of women who smoked more than 15 cigarettes in the last 24 hours	Women who smoke cigarettes
**Health**	HL_health_insur_p	Women being covered by health insurance (%)	-	
**Health – Birth level**	HL_IMR	Infant Mortality Rate (IMR)	See DHS.rates	See DHS.rates
**Health – Birth level**	HL_CMR	Child Mortality Rate (CMR)	See DHS.rates	See DHS.rates
**Health – Birth level**	HL_U5MR	Under-five Mortality Rate (U5MR)	See DHS.rates	See DHS.rates
**Health – Birth level**	HL_NNMR	Neonatal Mortality Rate (NNMR)	See DHS.rates	See DHS.rates
**Health – Birth level**	HL_PNNMR	Post-neonatal Mortality Rate (PNNMR)	See DHS.rates	See DHS.rates
**Reproductive health and fertility**	RH_children_born_mean	Average number of children ever born	See DHS.rates	See DHS.rates
**Reproductive health and fertility**	RH_children_living_mean	Average number of living children	See DHS.rates	See DHS.rates
**Fertility – complex indicators**	FF_TFR	Total Fertility Rate (based on a period of 3 year)	-	-
**Fertility – complex indicators**	FF_ASFR_15-19	Age Specific Fertility Rate (ASFR) for age period 15-19	-	-
**Fertility – complex indicators**	FF_ASFR_20-24	Age Specific Fertility Rate (ASFR) for age period 20-24	-	-
**Fertility – complex indicators**	FF_ASFR_25-29	Age Specific Fertility Rate (ASFR) for age period 25-29	-	-

Table 1: List of indicators based on DHS data present in the database. The indicators for which there is no information on the denominator and nominator were calculated based on the guide for DHS statistics. *(continued)*

Category	Indicator code	Indicator description	Nominator	Denominator or exposure
**Fertility – complex indicators**	FF_ASFR_30-34	Age Specific Fertility Rate (ASFR) for age period 30-34	-	-
**Fertility – complex indicators**	FF_ASFR_35-39	Age Specific Fertility Rate (ASFR) for age period 35-39	-	-
**Fertility – complex indicators**	FF_ASFR_40-44	Age Specific Fertility Rate (ASFR) for age period 40-44	-	-
**Fertility – complex indicators**	FF_ASFR_45-49	Age Specific Fertility Rate (ASFR) for age period 45-49	-	-
**Fertility – complex indicators**	FF_GFR	General Fertility Rate (GFR),	-	-
**Reproductive health and fertility**	RH_age_first_birth_mean	Mean age at first birth for women aged 25-49 who ever had a child	-	-
**Reproductive health and fertility**	RH_contr_p	Women currently using any method of contraception (%)	-	-
**Reproductive health and fertility**	RH_contr_modern_p	Women currently using modern contraception (%)	-	-
**Reproductive health and fertility**	RH_contr_modern_know_p	Women who know any modern method of contraception (%)	-	-
**Reproductive health and fertility**	RH_family_planning_tv_p	Women who heard about family planning on TV in the last few months (%)	-	-
**Reproductive health and fertility**	RH_family_planning_radio_p	Women who heard about family planning on radio in the last few months (%)	-	-
**Reproductive health and fertility**	RH_family_planning_news_p	Women who heard about family planning on newpaper in the last few months (%)	-	-
**Reproductive health and fertility**	RH_family_planning_mobile_p	Women who heard about family planning on a mobile phone in the last few months (%)	-	-
**Reproductive health and fertility**	RH_age_first_sex_mean	Average age at first sexual intercourse among women age 25-49 who had a sexual intercourse	-	-
**Fertility preferences**	FP_desired_mean	Average ideal number of children for all women	-	-
**Fertility preferences**	FP_desired_sons_mean	Average desired number of sons for all women	Not applicable (average)	Number of women who gave a numerical response
**Fertility preferences**	FP_desired_daugh_mean	Average desired number of daugthers for all women	Not applicable (average)	Number of women who gave a numerical response
**Household characteristics – Household level**	HH_time_water_mean	Average time to get to drinking water	Not applicable (average)	Households that do not have water on premise
**Household characteristics – Household level**	HH_toilet_high_p	Households with high quality toilet facility (%)	Number of households with high quality toilet facility (for the definition of toilet categories see Supplementary Table 5)	Number of households
**Household characteristics – Household level**	HH_toilet_low_p	Households with low quality toilet facility (%)	Number of households with low quality toilet facility (for the definition of toilet categories see Supplementary Table 5)	Number of households
**Household characteristics – Household level**	HH_floor_high_p	Households with high quality floor (%)	Number of households with high quality floor (for the definition of floor categories see Supplementary Table 4)	Number of households
**Household characteristics – Household level**	HH_floor_low_p	Households with low quality floor (%)	Number of households with low quality floor (for the definition of floor categories see Supplementary Table 4)	Number of households

Table 1: List of indicators based on DHS data present in the database. The indicators for which there is no information on the denominator and nominator were calculated based on the guide for DHS statistics. *(continued)*

Category	Indicator code	Indicator description	Nominator	Denominator or exposure
**Household characteristics – Household level**	HH_water_high_p	Households with high quality water access (%)	Number of households with high quality drinking water source (for the definition of drinking water source categories see Supplementary Table 6)	Number of households
**Household characteristics – Household level**	HH_water_low_p	Households with low quality water access (%)	Number of households with low quality drinking water source (for the definition of drinking water source categories see Supplementary Table 6)	Number of households
**Household characteristics – Household level**	HH_watch_p	Households with watch (%)	Number of households with a watch	Number of households
**Household characteristics – Household level**	HH_car_p	Households with car (%)	-	-
**Household characteristics – Household level**	HH_motorcycle_p	Households with motorcycle (%)	-	-
**Household characteristics – Household level**	HH_bicycle_p	Households with bicycle (%)	-	-
**Household demographics – Household level**	HD_size_dejure_mean	Average number of household members	-	-
**Household demographics – Household level**	HD_size_defacto_mean	Average number of household members	Not applicable (average)	All households
**Household demographics – Household level**	HD_children_mean	Average number of children resident in the household under the age of 5	Not applicable (average)	All households
**Energy and information – Household level**	EI_elec_p	Households with electricity (%)	-	-
**Energy and information – Household level**	EI_tv_p	Households with tv (%)	-	-
**Energy and information – Household level**	EI_radio_p	Households with radio (%)	-	-
**Energy and information – Household level**	EI_fridge_p	Households with fridge (%)	-	-
**Energy and information – Household level**	EI_telephone_p	Households with a telephone (%)	Number of households with a phone	Number of households
**Energy and information – Household level**	EI_modern_cooking_p	Households whose main cooking fuel is modern (%)	Number of households whose main type of cooking fuel is modern (for the definition of modern fuels see main manuscript)	Number of households
**Energy and information – Household level**	EI_computer_p	Households with computer (%)	-	-
**Energy and information – per urban/rural area**	ER_elec_rural_p	Households in rural areas with access to electricity (%)	Number of households with electricity access	Number of households living in rural areas
**Energy and information – per urban/rural area**	ER_elec_urban_p	Households in urban areas with access to electricity (%)	Number of households with electricity access	Number of households living in urban areas
**Household Wealth**	WL_wealth_mean	Average of the International Wealth Index	Not applicable (average)	All households
**Household Wealth**	WL_wealth_median	Median of the International Wealth Index	Not applicable (average)	All households

Table 1: List of indicators based on DHS data present in the database. The indicators for which there is no information on the denominator and nominator were calculated based on the guide for DHS statistics. *(continued)*

Category	Indicator code	Indicator description	Nominator	Denominator or exposure
**Household Wealth**	WL__wealth__gini	GINI coefficient of the International Wealth Index	Not applicable (average)	All households
**Nutrition**	NT__women__anemia__any__p	Women classified as having any anemia (<12.0 g/dl for non-pregnant women and <11.0 g/dl for pregnant women) (%)	Number of women classified as having any anemia (<12.0 g/dl for non-pregnant women and <11.0 g/dl for pregnant women)	Women whose hemoglobin level has been measured
**Socio-economic indicators**	hdi	Human Development Index		
**Socio-economic indicators**	gdp__pc	Gross Domestic Product (PPP) per capita in constant 2011 international US dollars		

Supplementary Table 2

Table 2: Overview of climate indicators based on high resolution gridded climate from CRU TS (4.05).

<table>
<tr><th>Category</th><th>Climate Indicator</th><th>Anomaly Threshold</th><th>Aggregation</th><th>Time period (months)</th><th>Interpretation</th><th>Example</th></tr>
<tr><td>Precipitation</td><td>pre</td><td>-</td><td>__mean</td><td>12 | 36 | 60</td><td>Mean monthly precipitation in mm in region over the time period</td><td>pre__mean36</td></tr>
<tr><td>Precipitation</td><td>pre</td><td>-</td><td>__max</td><td>12 | 36 | 60</td><td>Maximum monthly precipitation in mm in region over the time period</td><td>pre__max60</td></tr>
<tr><td>Precipitation</td><td>pre</td><td>-</td><td>__min</td><td>12 | 36 | 60</td><td>Minimum monthly precipitation in mm in region over the time period</td><td>pre__min12</td></tr>
<tr><td>Precipitation</td><td>pre</td><td>-</td><td>__sum</td><td>12 | 36 | 60</td><td>Sum of precipitation in mm in region over the time period</td><td>pre__sum12</td></tr>
<tr><td>Precipitation</td><td>pre__anom</td><td>__n1sd | __n1.5sd | __n2sd | __n3sd</td><td>__share</td><td>12 | 36 | 60</td><td>Share of months in time period with a negative precipitation anomaly exceeding threshold < 1SD, 1.5SD, 2SD, 3SD</td><td>pre__anom__n1.5sd__share36</td></tr>
<tr><td>Precipitation</td><td>pre__anom</td><td>__p1sd | __p1.5sd | __p2sd | __p3sd</td><td>__share</td><td>12 | 36 | 60</td><td>Share of months in time period with a positive precipitation anomaly exceeding threshold > 1SD, 1.5SD, 2SD, 3SD</td><td>pre__anom__p1sd__share12</td></tr>
<tr><td>Precipitation</td><td>pre__anom</td><td>-</td><td>__mean</td><td>12 | 36 | 60</td><td>Mean precipitation anomaly as the average monthly deviation from the long run precipitation mean</td><td>pre__anom__mean12</td></tr>
<tr><td>Precipitation</td><td>pre__anom</td><td>-</td><td>__abs__mean</td><td>12 | 36 | 60</td><td>Mean absolute precipitation anomaly as the average monthly absolute deviation from the long run precipitation mean</td><td>pre__anom__abs__mean36</td></tr>
<tr><td>Temperature</td><td>tmp</td><td>-</td><td>__mean</td><td>12 | 36 | 60</td><td>Mean monthly temperature in region over the time period</td><td>tmp__mean60</td></tr>
<tr><td>Temperature</td><td>tmp</td><td>-</td><td>__max</td><td>12 | 36 | 60</td><td>Maximum monthly temperature in region over the time period</td><td>tmp__max12</td></tr>
<tr><td>Temperature</td><td>tmp</td><td>-</td><td>__min</td><td>12 | 36 | 60</td><td>Minimum monthly temperature in region over the time period</td><td>tmp__min36</td></tr>
<tr><td>Temperature</td><td>tmp__anom</td><td>__n1sd | __n1.5sd | __n2sd | __n3sd</td><td>__share</td><td>12 | 36 | 60</td><td>Share of months in time period with a negative temperature anomaly exceeding threshold < 1SD, 1.5SD, 2SD. 3SD</td><td>tmp__anom__n1.5sd__share36</td></tr>
<tr><td>Temperature</td><td>tmp__anom</td><td>__p1sd | __p1.5sd | __p2sd | __p3sd</td><td>__share</td><td>12 | 36 | 60</td><td>Share of months in time period with a positive temperature anomaly exceeding threshold > 1SD, 1.5SD, 2SD. 3SD</td><td>tmp__anom__p1sd__share36</td></tr>
<tr><td>Temperature</td><td>tmp__anom</td><td>-</td><td>__mean</td><td>12 | 36 | 60</td><td>Mean temperature anomaly as the average monthly deviation from the long run temperature mean</td><td>tmp__anom__mean12</td></tr>
<tr><td>Temperature</td><td>tmp__anom</td><td>-</td><td>__abs__mean</td><td>12 | 36 | 60</td><td>Mean absolute temperature anomaly as the average monthly absolute deviation from the long run temperature mean</td><td>tmp__anom__abs__mean60</td></tr>
<tr><td>SPEI03</td><td>spei03</td><td>-</td><td>__mean</td><td>12 | 36 | 60</td><td>Mean monthly SPEI03 in region over the time period</td><td>spei03__mean60</td></tr>
</table>

Table 2: Overview of climate indicators based on high resolution gridded climate from CRU TS (4.05). *(continued)*

Category	Climate Indicator	Anomaly Threshold	Aggregation	Time period (months)	Interpretation	Example
**SPEI03**	spei03	-	__max	12 | 36 | 60	Maximum monthly SPEI03 in region over the time period	spei03__max12
**SPEI03**	spei03	-	__min	12 | 36 | 60	Minimum monthly SPEI03 in region over the time period	spei03__min36
**SPEI03**	spei03__anom	__n1sd | __n1.5sd | __n2sd | __n3sd	__share	12 | 36 | 60	Share of months in time period with a negative SPEI03 anomaly exceeding threshold < 1SD, 1.5SD, 2SD. 3SD	spei03__anom__n1.5sd__share36
**SPEI03**	spei03__anom	__p1sd | __p1.5sd | __p2sd | __p3sd	__share	12 | 36 | 60	Share of months in time period with a positive SPEI03 anomaly exceeding threshold > 1SD, 1.5SD, 2SD. 3SD	spei03__anom__p1sd__share12
**SPEI03**	spei03__anom	-	__mean	12 | 36 | 60	Mean SPEI03 anomaly as the average monthly deviation from the long run SPEI03 mean	spei03__anom__mean12
**SPEI03**	spei03__anom	-	__abs__mean	12 | 36 | 60	Mean absolute SPEI03 anomaly as the average monthly absolute deviation from the long run SPEI03 mean	spei03__anom__abs__mean60
**Drought**	spei03	__n1 | __n1.5 | __n2	__share	12 | 36 | 60	Share of months in time period with drought conditions (SPEI < threshold)	drought__spei03__n1__share12

Supplementary Table 3

Table 3: Variables used to calculate the International Wealth Index and the corresponding first factor of the Principal Component Analysis derived by Smits et al 2015

Variable	Value of the first factor of the PCA
Whether the household has a TV	8.612657
Whether the household has a fridge	8.429076
Whether the household has a telephone	7.127699
Whether the household has a car	4.651382
Whether the household has a bicycle	1.846860
Whether the household has a low quality floor	-7.558471
Whether the household has a medium quality floor	1.227531
Whether the household has a high quality floor	6.107428
Whether the household has a low quality toilet	-7.439841
Whether the household has a medium quality toilet	-1.090393
Whether the household has a high quality toilet	8.140637
Whether the household has a low quality source of drinking water	-6.306477
Whether the household has a medium quality source of drinking water	-2.302023
Whether the household has a high quality source of drinking water	7.952443
Whether the household has a 1 sleeping rooms	-3.699681
Whether the household has a 2 sleeping rooms	0.384050
Whether the household has a more than three sleeping rooms	3.445009
Whether the household has access to electricity	8.056664
Whether the household has an expensive utensil (computer, motocycle or car)	4.118394
Whether the household has a cheap utensil (watch or having an expensive utensil).	6.507283
Nominator	25.004470

Supplementary Table 4, 5 and 6

Table 4: All labels for type of floor found in DHS surveys and the corresponding assigned category

**Type of floor category**	**Type of floor**
**High quality**	Parquet; Polished wood; Lynoleum; Carpet; Vinyl, Asphalt strips; Ceramic or marble tiles; Cement tiles; Rubber, vinyl; Marmol; Rug, carpet; Terazzo; Mosaic; PVC; Mats; Karo
**Low quality**	Earth; Sand; Dung; Dirt; Clay; Soil; Mud mixed with dung
**Middle quality**	Wood planks; Cement; Palm/Bamboo; Rudimentary floor - Wood planks; Cement ; Earth/Bamboo; Bricks;
**NA**	Not dejure resident; Other; Not a dejure resident

Table 5: All labels for type of toilet found in DHS surveys and the corresponding assigned category

**Type of toilet category**	**Type of toilet**
**High quality**	Flush Toilet; Flush - to piped sewer system; Flush - to septic tank; Flush - to pit latrine; Flush toilet connected to sewer system; Flush toilet not connected to sewer system; Private flush toilet; Shared flush toilet; Flush toilet (private or shared unknown) Flush connected to sewer/with septic tank; Flush unconnected to sewer/without septic tank; Modern flush toilet; Traditional with tank flush; Modern flush toilet; Traditional tank flush; Traditional bucket flush
**Low quality**	No facility, bush, field; Pit latrine without slab; Open pit; Latrine, Bucket toilet; Hanging toilet;Traditional Pit/Latrine unconnected to sewer/without septic;
**Middle quality**	Public Flush Toilet; Pit latrine - ventilated improved pit ; Pit latrine - with slab; Composting toilet; Bucket, pan; Bucket/pan toilet; Public connected to sewer; Covered latrine; Ventilated improved pit latrine; ....Pit latrine - ventilated improved; ....Pit latrine - with slab; Latrine with composting facility; Closed pit; Ecosan; Blair toilet
**NA**	Not a dejure resident

Table 6: All labels for source of drinking water found in DHS surveys and the corresponding assigned category

Drinking water category	Type of drinking water
**High quality**	Piped into residence; Piped into yard/plot; Bottled water; Piped - into dwelling; Public tap/standpipe; Pipe into dwelling (own artesian); Piped to neighbor; Public tap/standpipe; Sachet water; Water from vendor
**Low quality**	Open well in residence; Open well in yard/plot; Spring; River; stream; Pond; Lake; Dam; Rainwater; Surface water Nile; Canal; Irrigation water
**Middle quality**	Piped - public tap / standpipe; Public piped network; Public water piped outside the household; Piped - public tap / standpipe; Piped public /private; Public piped water outside house/garden; Public tap; Tube well or borehole; Protected well to yard; Water from neighbors; Neighbor's house;
**NA**	Not dejure resident;

Supplementary Table 7

Table 7: Information on the temporal dimensions and the region harmonization of countries represented in LivWell

Country Name	Number of waves	First year	Last year	Average wave gap	Number of observations	Harmonized regions
Armenia	4	2000	2016	5.3	44	no
Bangladesh	8	1994	2017	3.3	40	yes
Benin	5	1996	2017	5.2	30	yes
Bolivia	3	1998	2008	5.0	24	yes
Burkina Faso	2	2003	2010	7.0	26	yes
Burundi	2	2010	2016	6.0	8	yes
Cambodia	4	2000	2014	4.7	72	yes
Cameroon	3	2004	2018	7.0	22	yes
Colombia	6	1990	2015	5.0	33	yes
Congo Democratic Republic	2	2007	2013	6.0	22	no
Cote d'Ivoire	2	1994	2012	18.0	20	yes
Egypt	7	1992	2014	3.7	25	yes
Ethiopia	4	2000	2016	5.3	44	no
Gabon	2	2000	2012	12.0	10	yes
Ghana	5	1993	2014	5.2	50	yes
Guatemala	3	1995	2015	10.0	23	yes
Guinea	3	2005	2018	6.5	24	yes
Haiti	4	2000	2016	5.3	36	yes
Honduras	2	2005	2011	6.0	32	yes
India	3	1993	2006	6.5	76	yes
Indonesia	6	1994	2017	4.6	155	yes
Jordan	5	1997	2017	5.0	15	yes
Kenya	5	1993	2014	5.2	38	yes
Lesotho	3	2004	2014	5.0	30	no
Liberia	2	2007	2013	6.0	10	yes
Madagascar	4	1992	2008	5.3	24	yes
Malawi	5	1992	2015	5.8	15	no
Maldives	2	2009	2016	7.0	12	no
Mali	5	1996	2018	5.5	37	yes
Morocco	2	1992	2003	11.0	12	yes
Mozambique	3	1997	2011	7.0	33	no
Namibia	3	2000	2013	6.5	39	no
Nepal	3	1996	2006	5.0	15	no
Nicaragua	2	1998	2001	3.0	34	no
Niger	4	1992	2012	6.7	24	yes
Nigeria	4	2003	2018	5.0	24	no
Pakistan	4	1991	2017	8.7	17	yes
Peru	8	1996	2012	2.3	192	yes
Philippines	4	2003	2017	4.7	68	yes
Rwanda	3	2008	2015	3.5	15	no
Senegal	11	1993	2019	2.6	44	yes
Sierra Leone	3	2008	2019	5.5	12	yes
South Africa	2	1998	2016	18.0	8	yes
Tajikistan	2	2012	2017	5.0	10	no
Tanzania	5	1996	2015	4.8	103	yes
Timor-Leste	2	2009	2016	7.0	26	no
Togo	2	1998	2013	15.0	12	no
Turkey	3	2003	2013	5.0	15	yes
Uganda	5	1995	2016	5.2	20	yes
Vietnam	2	1997	2002	5.0	14	no
Zambia	6	1992	2018	5.2	48	yes
Zimbabwe	5	1994	2015	5.2	50	yes

Supplementary Table 8, 9 and 10

Table 8: Summary statistics for all DHS-based indicators in LivWell (Part 1)

Statistic	N	Mean	St. Dev.	Min	Pctl(25)	Pctl(75)	Max
interview_year_mean	1,824	2,005.7	7.3	1,990.0	2,000.0	2,012.0	2,019.0
interview_month_mean	1,824	7.5	3.3	1.0	5.0	10.0	13.0
CMC_interview_mean	1,824	1,276.2	87.4	1,085.4	1,204.1	1,347.3	1,436.5
DM_age_mean	1,824	28.2	2.4	17.7	27.7	29.7	33.6
DM_age_15-19_p	1,824	18.3	7.0	0.4	16.5	22.9	33.2
DM_age_20-24_p	1,824	16.9	3.7	4.8	15.0	19.2	34.3
DM_age_25-29_p	1,824	16.0	2.4	8.8	14.5	17.6	27.7
DM_age_30-34_p	1,824	13.8	1.8	7.7	12.6	15.0	21.1
DM_age_35-39_p	1,824	12.2	1.9	1.3	11.0	13.4	19.0
DM_age_40-44_p	1,824	10.1	2.1	1.8	8.6	11.5	17.8
DM_age_45-49_p	1,824	8.5	2.3	2.6	6.9	10.0	18.9
DM_urban_p	1,824	36.8	26.4	0.0	16.7	53.1	100.0
DM_born_rural_p	859	56.3	24.9	0.0	34.8	77.3	97.7
DM_nvr_marr_p	1,534	27.7	9.6	2.7	21.6	33.3	77.0
DM_marr_p	1,824	64.6	10.3	19.6	58.0	70.8	96.1
DM_age_marr_mean	1,827	18.8	1.7	13.5	17.7	20.0	26.8
DM_age_diff_mean	1,620	6.0	2.7	1.4	4.0	7.5	16.4
DM_age_diff_10plus_p	1,620	21.5	15.1	0.0	11.3	26.7	100.0
DM_age_diff_5_9_p	1,620	33.1	10.1	0.0	27.7	39.7	64.8
DM_age_diff_5minus_p	1,620	34.5	15.5	0.0	22.4	46.6	75.5
DM_age_diff_0_p	1,620	3.6	3.4	0.0	0.8	5.7	18.5
HH_women_time_water_mean	1,686	22.5	15.6	1.4	12.3	28.8	247.5
HH_women_toilet_high_p	1,725	26.1	28.4	0.0	2.1	43.4	100.0
HH_women_toilet_low_p	1,725	51.5	29.6	0.0	27.7	76.6	100.0
HH_women_water_high_p	1,756	34.3	30.6	0.0	7.7	58.9	100.0
HH_women_water_low_p	1,756	27.6	22.7	0.0	8.7	41.6	100.0
HH_women_floor_high_p	1,627	16.7	20.7	0.0	1.6	25.0	99.0
HH_women_floor_low_p	1,627	35.5	29.7	0.0	7.7	60.6	99.7
HD_women_size_mean	1,824	6.1	1.8	3.0	5.1	6.6	15.2
HD_women_children_mean	1,824	1.0	0.5	0.2	0.7	1.2	3.3
WK_working_p	1,765	51.7	20.7	0.0	37.7	67.7	100.0
WK_working12_p	1,663	57.9	20.5	2.7	44.0	74.5	99.1
WK_working_paid_p	1,382	59.2	24.0	2.0	41.2	78.8	99.2
WK_working_agri_p	1,620	36.2	26.3	0.0	12.5	56.7	97.0
ED_educ_years_mean	1,824	5.7	2.9	0.2	3.5	8.0	12.9
ED_attainment_no_educ_p	1,824	23.5	24.7	0.0	3.7	35.5	94.9
ED_attainment_primary_p	1,824	32.7	18.8	0.0	17.6	46.0	83.4
ED_attainment_primary_completed_p	1,813	12.6	12.0	0.0	4.4	16.6	68.3
ED_litt_p	1,769	74.9	26.3	3.6	59.7	96.7	100.0
ED_litt_whole_p	1,253	59.1	27.5	2.6	38.5	85.7	99.2
EI_women_elec_p	1,768	48.9	32.8	0.0	17.0	78.8	100.0
EI_women_radio_p	1,809	56.8	22.9	0.2	39.4	75.9	98.2
EI_women_tv_p	1,821	43.0	30.6	0.0	14.4	69.1	100.0
EI_women_fridge_p	1,777	24.0	24.4	0.0	4.0	38.8	99.8
EI_women_telephone_p	1,545	12.0	16.0	0.0	1.4	17.0	96.0
EI_women_modern_cooking_p	1,365	29.0	31.3	0.0	1.6	50.6	100.0
EI_mobile_p	216	63.2	20.5	10.1	50.9	80.0	99.4
EI_internet_week_p	211	24.8	22.8	0.7	5.4	40.7	89.2
EI_internet_day_p	211	17.4	18.3	0.3	3.0	26.7	78.2
EI_news_week_p	1,359	16.9	14.0	0.3	6.1	23.5	74.7

Table 9: Summary statistics for all DHS-based indicators in LivWell (Part 2)

Statistic	N	Mean	St. Dev.	Min	Pctl(25)	Pctl(75)	Max
EI_radio_week_p	1,354	46.9	23.0	0.2	27.6	65.2	93.5
EI_tv_week_p	1,354	45.4	27.9	0.8	19.2	69.8	99.9
DP_decide_money_p	1,375	52.5	21.4	0.5	35.4	70.0	100.0
DP_decide_health_p	1,332	31.2	19.3	0.1	15.2	44.1	92.8
DP_decide_large_purchase_p	1,337	15.2	9.5	0.3	8.1	20.3	81.1
DP_decide_visits_p	510	67.9	13.8	13.0	60.8	77.4	93.6
DP_owns_house_p	539	38.1	20.1	1.1	23.8	51.5	98.4
DP_owns_land_p	519	29.8	19.4	0.4	14.1	43.1	98.0
DP_decide_contraception_p	222	23.6	14.9	0.0	12.7	31.7	84.0
DP_decide_no_contraception_p	206	33.9	17.4	1.2	21.7	43.7	76.9
DP_earn_more_equal_p	936	30.4	13.5	1.0	21.8	37.3	86.3
DP_earn_more_p	936	13.0	6.5	0.7	8.5	16.6	48.3
DV_phys_partner_p	809	29.0	14.3	0.0	16.5	40.2	77.0
DV_phys_partner_12m_p	809	18.6	12.4	0.0	9.7	24.4	73.7
DV_phys_p	825	33.2	14.4	0.0	22.6	43.3	79.7
DV_phys_12m_p	809	16.2	10.5	0.0	8.5	21.5	66.9
HL_smoke_p	1,232	4.4	6.0	0.0	0.8	5.7	60.8
HL_smoke_cig_p	1,227	2.7	5.2	0.0	0.3	3.5	63.0
HL_number_cigarettes_mean	1,082	14.0	20.5	0.0	3.1	13.7	99.0
HL_smoke5_p	1,061	30.0	26.7	0.0	6.7	47.2	100.0
HL_smoke10_p	1,034	15.8	19.8	0.0	0.0	24.4	100.0
HL_smoke15_p	1,024	7.7	14.2	0.0	0.0	10.4	100.0
HL_health_insur_p	732	20.7	24.0	0.0	2.1	37.3	91.2
RH_children_born_mean	1,824	2.5	0.6	0.9	2.0	2.9	4.9
RH_children_living_mean	1,824	2.2	0.5	0.9	1.9	2.5	3.8
RH_age_first_birth_mean	1,824	20.0	1.3	16.5	19.0	21.1	23.9
RH_contr_p	1,824	30.9	15.0	0.1	18.5	43.8	69.1
RH_contr_modern_p	1,824	24.5	13.7	0.1	13.2	35.3	65.7
RH_contr_modern_know_p	1,809	89.0	13.7	23.1	82.3	98.9	100.0
RH_family_planning_tv_p	1,676	30.7	21.9	0.0	11.9	46.5	94.5
RH_family_planning_radio_p	1,676	36.6	19.6	0.0	20.0	50.3	96.8
RH_family_planning_news_p	1,657	16.5	13.8	0.2	5.4	25.0	72.1
RH_family_planning_mobile_p	489	14.4	14.6	0.0	3.4	21.8	76.3
FP_desired_mean	1,824	3.7	1.6	1.4	2.5	4.8	16.6
FP_desired_sons_mean	1,703	1.6	0.8	0.3	1.0	2.1	13.2
FP_desired_daugh_mean	1,703	1.6	0.8	0.3	1.0	2.0	13.1
NT_women_anemia_any_p	930	37.3	16.1	5.6	22.9	50.6	74.7
FF_TFR	1,832	4.1	1.5	0.9	2.9	5.3	8.7
FF_ASFR_15-19	1,808	96.1	51.6	4.1	54.7	130.0	296.9
FF_ASFR_20-24	1,808	199.5	63.3	52.7	149.9	246.1	397.5
FF_ASFR_25-29	1,808	195.5	62.7	27.4	145.5	242.3	387.8
FF_ASFR_30-34	1,808	160.9	65.6	6.7	110.5	209.6	413.9
FF_ASFR_35-39	1,808	111.2	57.8	0.0	66.3	151.9	376.5
FF_ASFR_40-44	1,808	51.1	35.5	0.0	22.5	74.9	291.2
FF_ASFR_45-49	1,808	15.1	19.4	0.0	0.0	21.7	174.6
FF_GFR	1,832	142.1	51.7	34.3	101.5	180.4	302.2
HH_time_water_mean	1,696	23.0	15.2	1.6	12.9	29.0	249.3
HH_toilet_high_p	1,756	26.3	29.9	0.0	1.7	42.6	100.0
HH_toilet_low_p	1,756	54.4	30.0	0.0	30.3	80.5	100.0
HH_water_high_p	1,761	34.6	31.1	0.0	7.5	59.7	100.0

Table 10: Summary statistics for all DHS-based indicators in LivWell (Part 3)

Statistic	N	Mean	St. Dev.	Min	Pctl(25)	Pctl(75)	Max
HH_water_low_p	1,761	30.6	24.7	0.0	10.0	46.1	100.0
HH_floor_high_p	1,754	16.5	22.1	0.0	1.2	22.8	98.6
HH_floor_low_p	1,754	39.3	31.3	0.0	9.6	66.6	100.0
HH_car_p	1,766	7.2	8.8	0.0	1.6	9.2	63.7
HH_motorcycle_p	1,773	14.1	18.5	0.0	1.5	19.9	90.2
HH_bicycle_p	1,791	27.6	20.0	0.0	12.0	39.3	93.7
HH_watch_p	606	39.9	20.9	4.1	22.2	53.9	97.5
HD_size_dejure_mean	1,832	4.9	1.1	2.9	4.2	5.3	10.4
HD_size_defacto_mean	1,778	4.8	1.1	2.8	4.1	5.2	10.5
HD_children_mean	1,832	0.8	0.3	0.2	0.6	1.0	2.3
EI_elec_p	1,776	49.4	34.4	0.0	15.2	82.7	100.0
EI_radio_p	1,817	55.4	21.7	0.4	39.4	73.1	97.6
EI_tv_p	1,829	40.9	30.7	0.0	12.0	66.8	99.9
EI_fridge_p	1,785	22.6	25.2	0.0	3.2	34.3	99.3
EI_telephone_p	1,553	11.6	16.5	0.0	1.1	15.2	95.4
EI_computer_p	222	15.8	18.2	0.1	3.1	21.9	86.8
EI_modern_cooking_p	1,373	30.5	32.9	0.0	2.0	52.8	100.0
ER_elec_rural_p	1,701	36.4	34.1	0.0	4.5	66.1	100.0
ER_elec_urban_p	1,761	72.8	28.2	0.0	51.8	96.4	100.0
WL_wealth_mean	1,832	37.5	19.0	6.2	22.1	51.0	89.2
WL_wealth_median	1,832	35.4	21.5	0.0	17.3	52.4	94.0
WL_wealth_gini	1,832	0.4	0.1	0.1	0.3	0.5	0.9
HL_NNMR	1,802	26.4	13.9	0.0	16.0	34.8	90.7
HL_PNNMR	1,802	25.4	19.8	0.0	10.8	34.4	136.1
HL_IMR	1,802	51.9	30.1	0.0	28.8	69.5	197.2
HL_CMR	1,802	30.6	32.2	0.0	8.4	41.9	262.7
HL_U5MR	1,802	80.1	54.8	0.0	37.6	109.3	360.1
